# Supplementary material for: Dissecting cell-free DNA fragmentation variation in tumors using cell line-derived xenograft mouse
Source: PLoS One. 2025 Jul 15;20(7):e0327483. doi: 10.1371/journal.pone.0327483 (PMC12262859; doi:10.1371/journal.pone.0327483)
Supplement: S1 Text — (DOCX) [file pone.0327483.s001.docx]

**Supplementary figures**

**
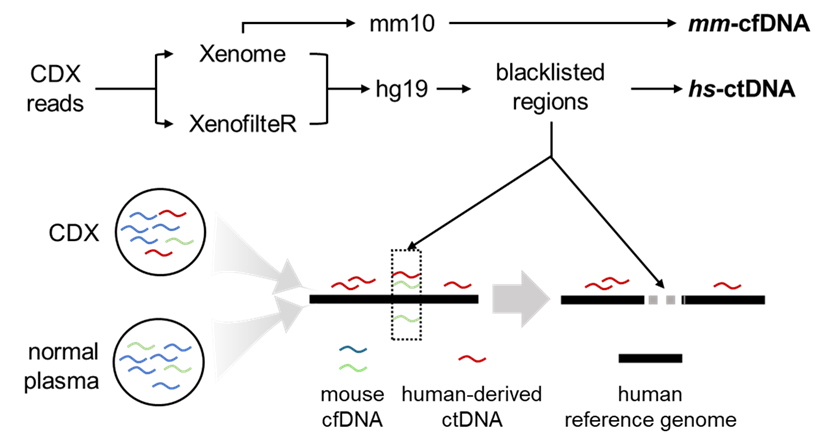
**

**Figure 1. A bioinformatic pipeline to isolate pure human-derived ctDNA from the xenograft mouse model, including the establishment of the blacklisted regions.**

**
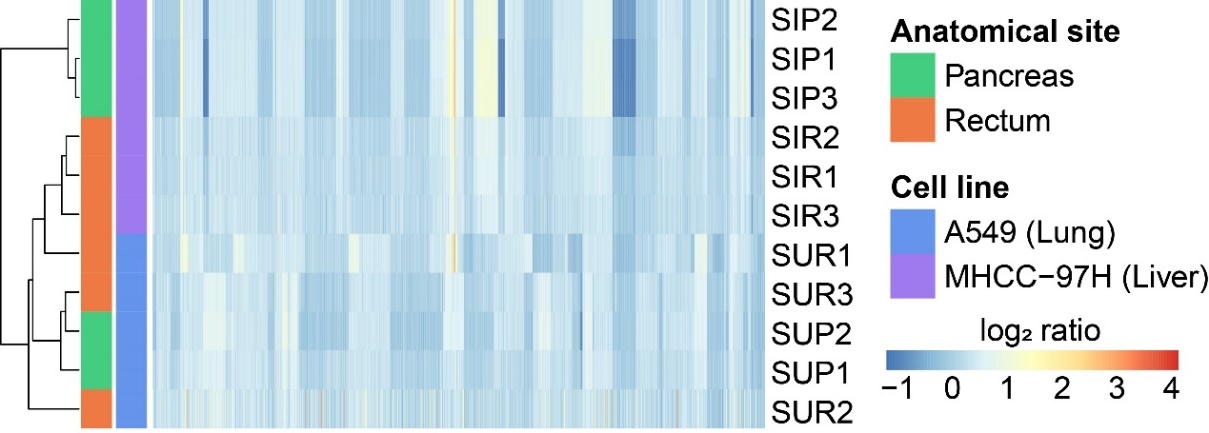
**

**Figure 2. Hierarchical clustering of copy number alteration (CNA) patterns of CDX** **samples, without SUP3.**

**
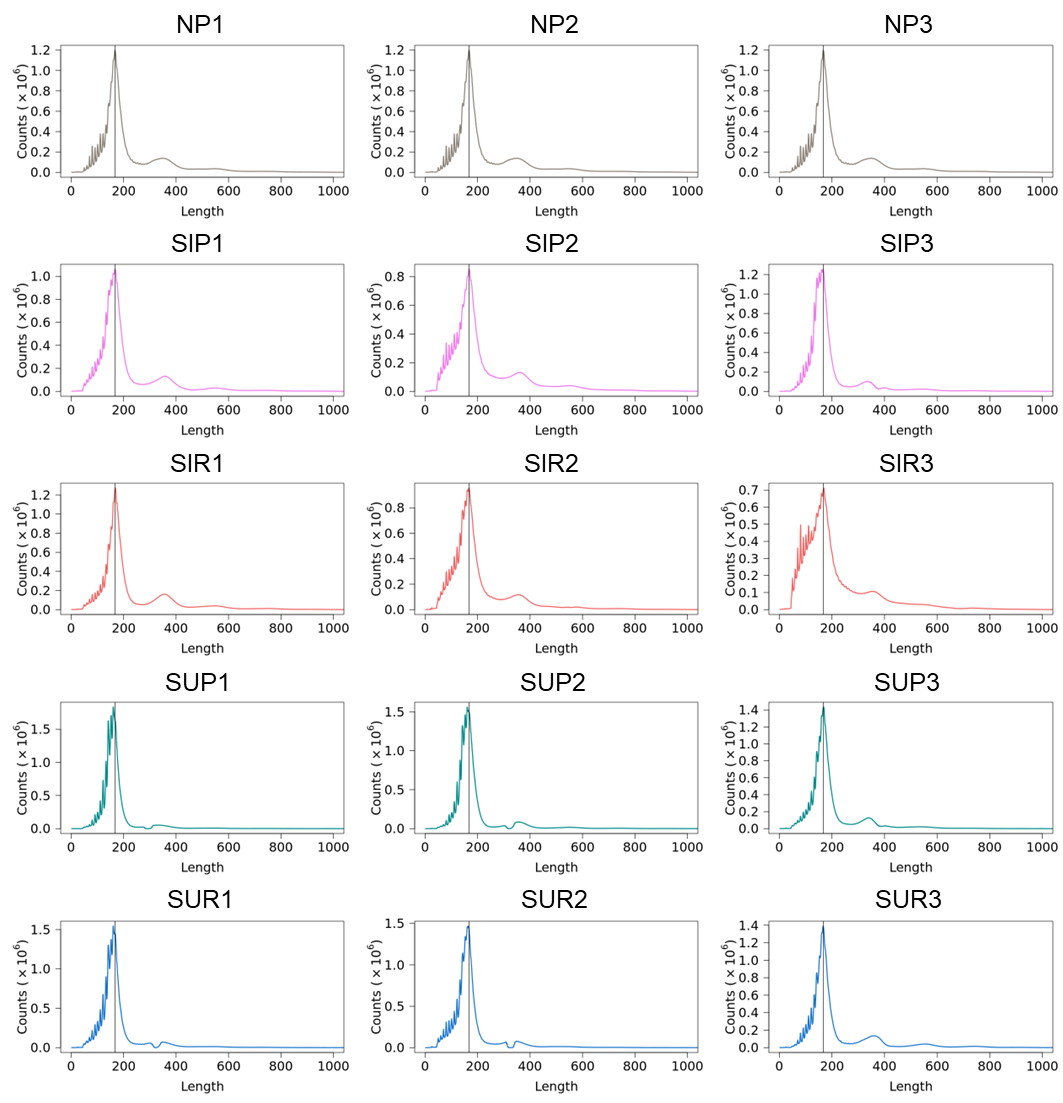
**

**Figure 3. Fragment size distribution of individual mouse plasma cfDNA, with a mode size of ~167 bp.**


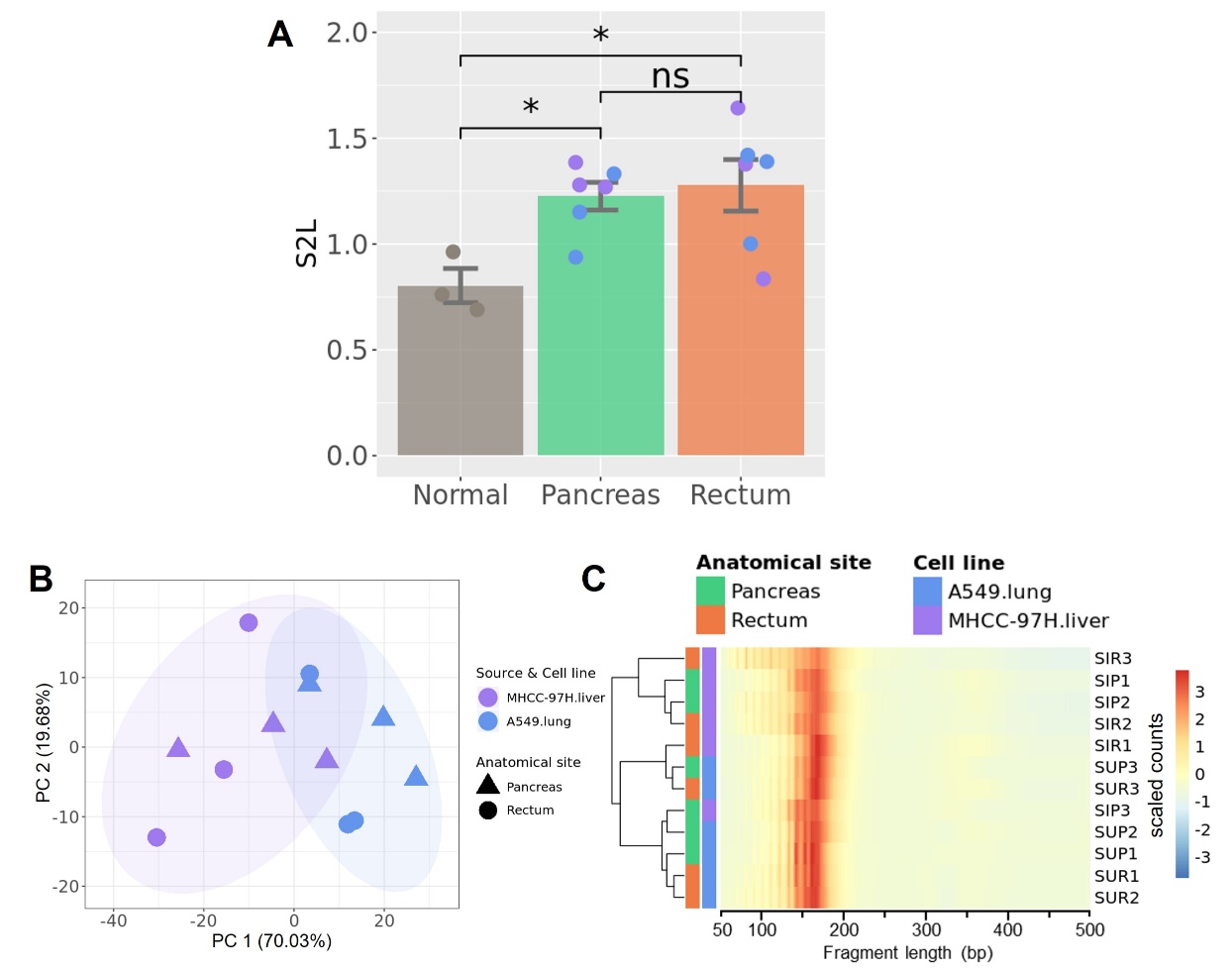


**Figure 4. Enrichment of short fragments in CDX-cfDNA from CDX models, indicated by S2L ratio, with points representing samples and CDX models grouping by anatomical sites (A). The p-values and 95% CI of U-test between normal and pancreas/rectum samples are 0.048/0.048 and [-0.641, -0.178]/[-0.880, -0.042]. Principal component analysis (PCA), displaying the variance explained by each PC, with colored ovals represent 95% confidence ellipses for the corresponding groups (B) and hierarchical clustering (C) of the fragment sizes of xenograft mouse plasma cfDNA exhibit differentiations between CDX models implanted with different tumor cell lines.**


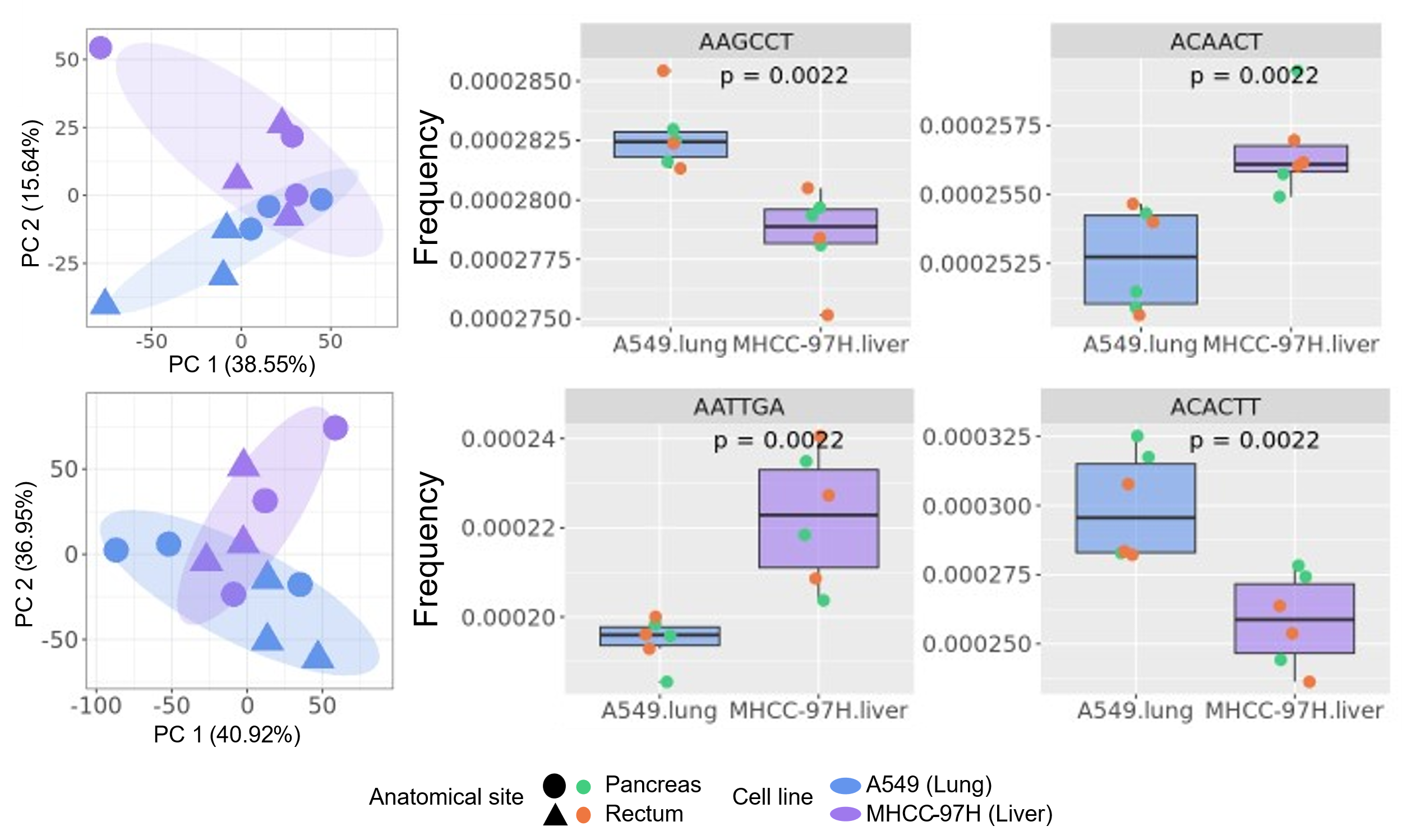


**Figure 5. CDX models of different cell lines are distinguished mainly by CDX-cfDNA fragmentation feature of breakpoint motif (BPM) (top) and end motif (EDM) (bottom), with several cases of the significantly differentiated motifs shown. PCA plots display the variance explained by each PC, and colored ovals represent 95% confidence ellipses for the corresponding groups. P values calculated by U-test.**


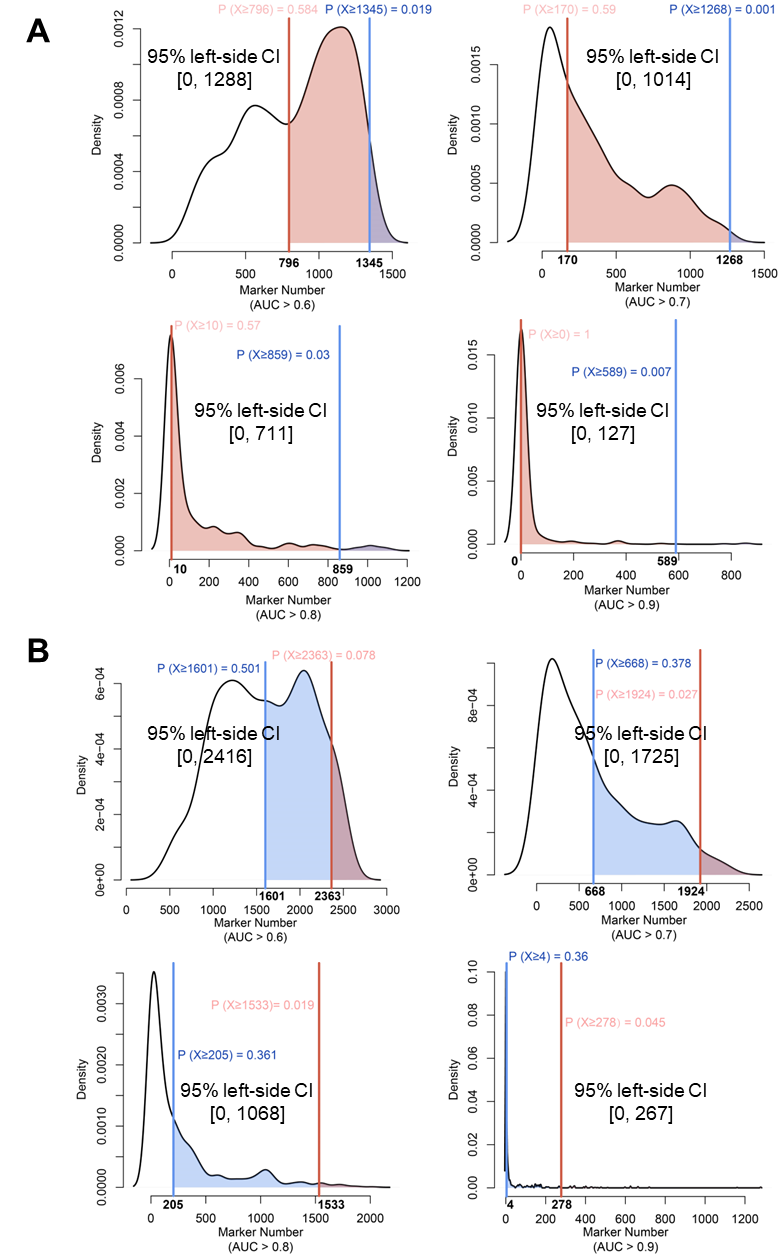


**Figure** **6.** **Distribution of informative markers (defined by different cut-offs of AUC) for FSD feature, based on 1,000 permutations and calculated for CDX-cfDNA (A) and ctDNA (B). The observed informative marker numbers for distinguishing between different cell lines (blue) and different anatomical sites (red) are shown by vertical lines. The calculation of the p value of permutation test is shown for each observed informative marker count. The 95% left-side CI of the informative marker count based on permutations is also shown.**


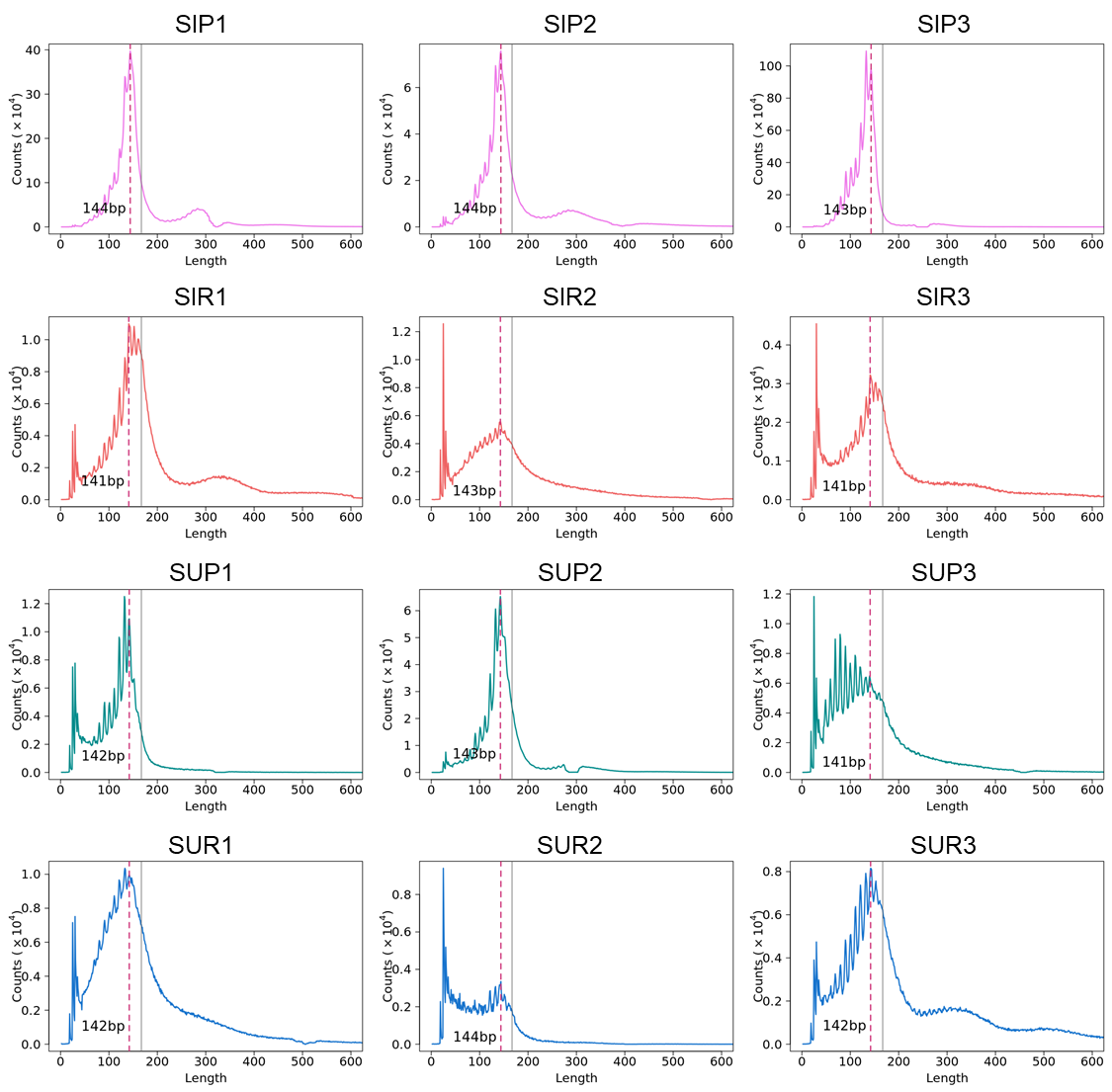


**Figure 7. Fragment size distribution of individual ctDNA from CDX models, with a mode size of ~143 bp.**


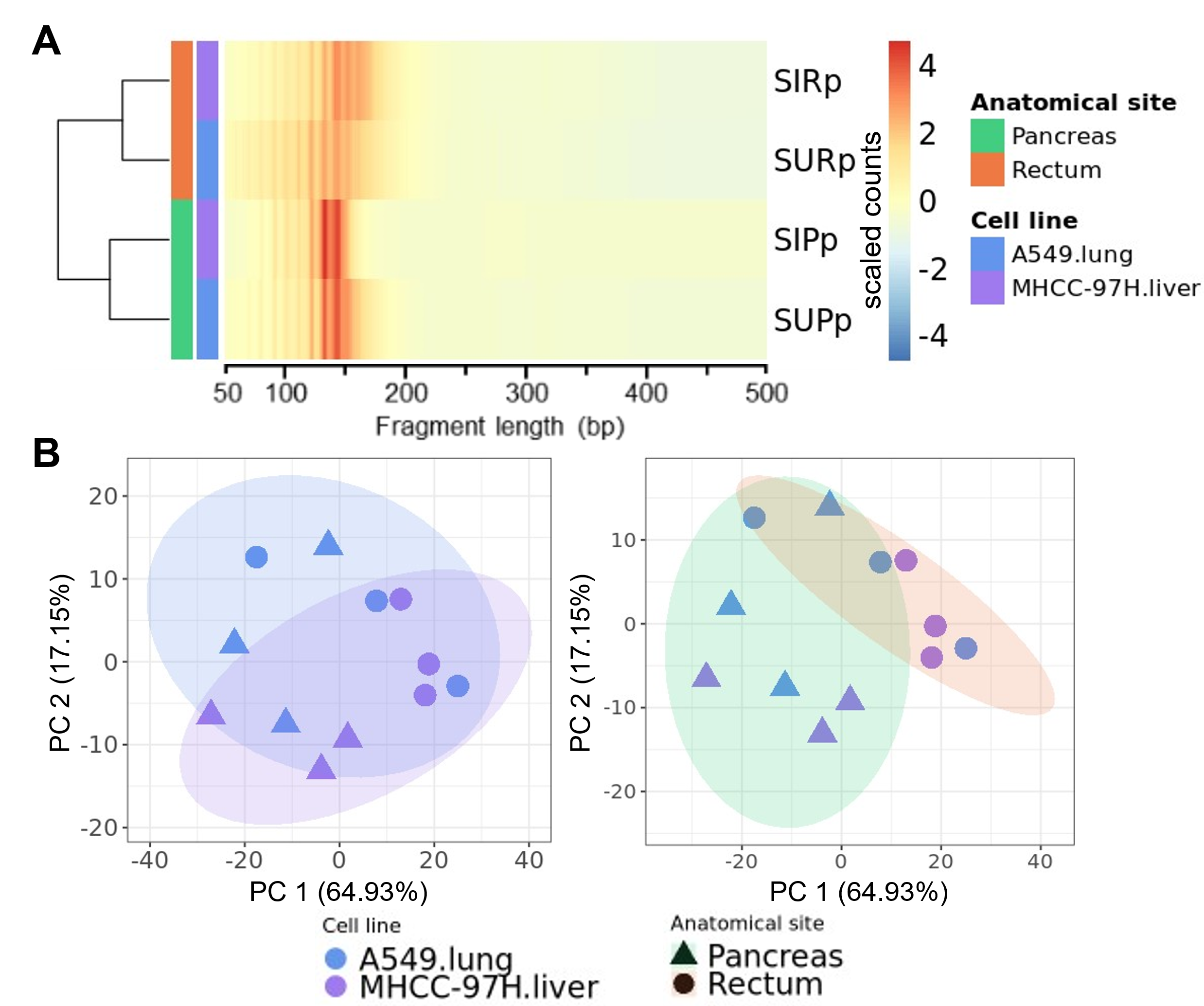


**Figure 8. (A) Hierarchical clustering by the fragment sizes of ctDNA from pooled representative samples. (B) PCA by fragment sizes of ctDNA from CDX models, grouped by different cell lines (left) and by different anatomical sites (right), displaying the variance explained by each PC. The colored ovals represent 95% confidence ellipses for the corresponding groups.**


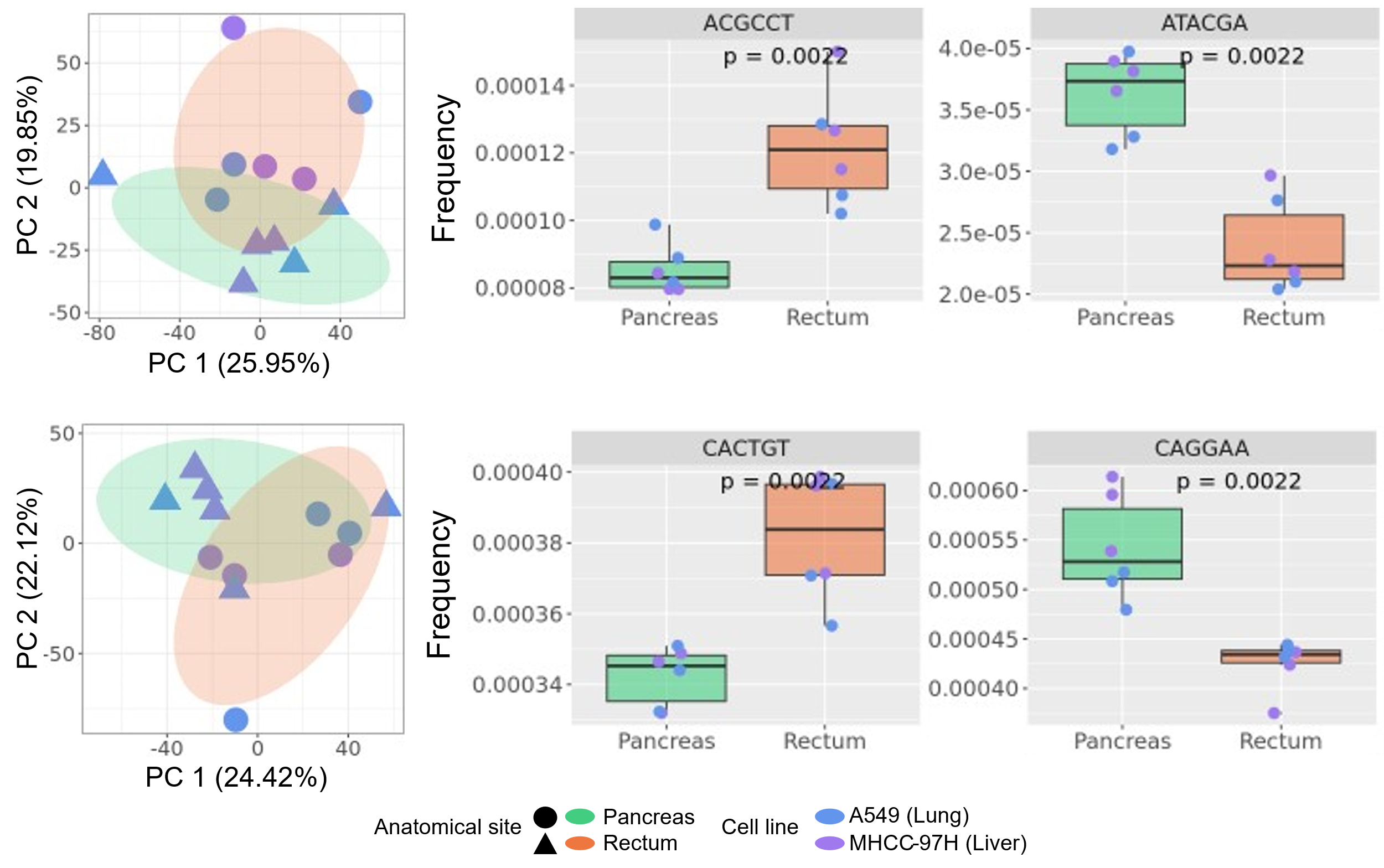


**Figure 9. CDX models of different anatomical sites are distinguished mainly by ctDNA fragmentation feature of BPM and EDM, with several cases of the significantly differentiated motifs shown. PCA plots display the variance explained by each PC, and colored ovals represent 95% confidence ellipses for the corresponding groups. P values calculated by U-test.**


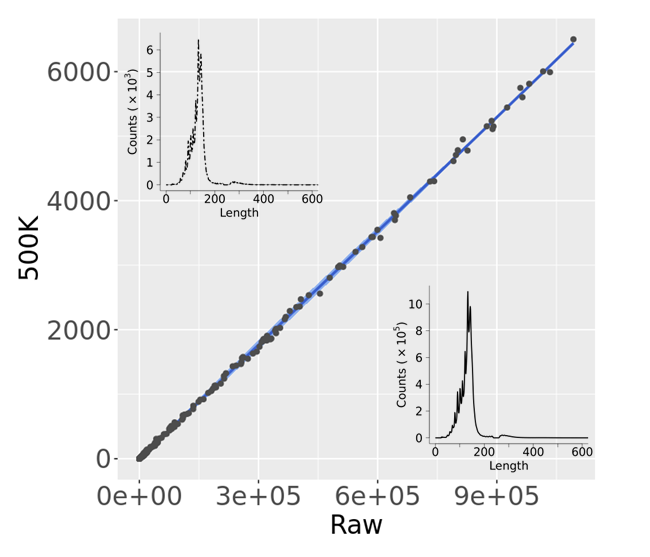


**Figure 10. Correlation of fragmentation profiles between raw ctDNA data and down-sampled ctDNA data (~500K reads), with the inset plots showing the corresponding fragment size distributions.**


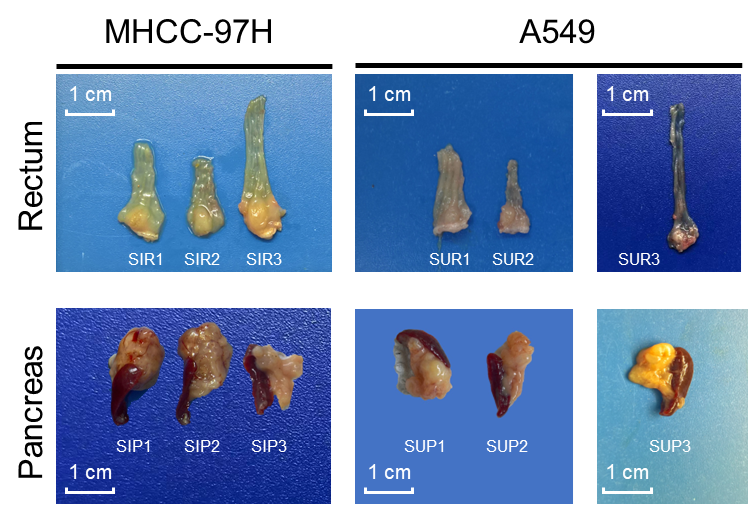


**Figure 11. Images of mouse organs implanted with tumors. Scale bars and sample identifiers are annotated in the figure. Background removal was applied to some of the samples to enhance the visibility of the organs with tumors, with no additional image processing performed.**
